# Improvement of Asymmetric Vestibulo-Ocular Reflex Responses Following Onset of Vestibular Neuritis Is Similar Across Canal Planes

**DOI:** 10.3389/fneur.2020.565125

**Published:** 2020-10-06

**Authors:** John H. J. Allum, Flurin Honegger

**Affiliations:** Division of Audiology and Neurootology, Department of Oto-rhino-laryngology, University of Basel Hospital, Basel, Switzerland

**Keywords:** vestibular neuritis (VN), vestibulo-ocular reflex (VOR), central compensation, peripheral recovery, vHIT

## Abstract

**Background:** We examined whether, after onset of acute unilateral vestibular neuritis (aUVN), initial disease effects, subsequent peripheral recovery and central compensation cause similar changes in vestibular ocular reflex (VOR) gains in all 3 semi-circular canal planes.

**Methods:** 20 patients, mean age 56.5 years, with pathological lateral canal video head impulse test (vHIT) VOR gains due to aUVN, were subsequently examined with vHIT in all 3 canal planes on average 4.3 and 36.7 days (“5 weeks”) after aUVN onset.

**Results:** Lateral and anterior deficit side (DS) average gains equaled 0.41 at aUVN onset. Non-deficit, normal, side (NS) gains were 0.88 and 0.81, respectively. Mean posterior DS gain was similar at onset, 0.43, provided only gains lower than 0.6 (lower limit of healthy controls) were considered. NS posterior mean gain at onset (0.68) was less (*p* ≤ 0.0006) than lateral and anterior NS gains. After 5 weeks, DS lateral, anterior and posterior canal gains increased (*p* ≤ 0.05), on average, to 0.65, 0.59, and 0.58, respectively. NS gains increased to 0.91, 0.87, and 0.76 (*p* = 0.007), respectively. At 5 weeks deficit-lateral/normal-lateral canal plane gain asymmetries were significantly (*p* < 0.0008) reduced from 36.9 to 19.4%, deficit-anterior/normal-posterior asymmetry decreased from 28.6 to 18.1%, while deficit-posterior/normal-anterior asymmetry changed from 29.7 to 21.4%, all to circa 20%. Roll plane asymmetries decreased slightly over 5 weeks (28.6–18.1%) but pitch plane asymmetries remained significantly less (*p* = 0.001), not different from 0% regardless of initial DS posterior canal vHIT gain. Yaw plane asymmetry changes are identical to those of the lateral canals (36.7–19.4%).

**Conclusions:** These results indicate that, at onset, aUVN of the superior vestibular nerve has a similar effect on lateral and anterior deficit DS VOR gains, and on posterior DS canal VOR gains if the inferior nerve was also affected at onset. The significant improvements to equal 5 week levels of DS gains and slightly greater posterior NS gain improvements, compared to lateral and anterior NS gains, yielding a common canal plane gain asymmetry of 20% at 5 weeks, suggest similar neural compensation mechanisms were active along VOR pathways. Unexpectantly, canal plane improvement was not replicated in pitch plane asymmetries.

## Introduction

With the introduction of the vHIT technique for testing vertical semi-circular canal function (1), it became possible to investigate whether weaknesses in any of the 6 directions of canal gains cause corresponding weaknesses in balance control for one or more of the 3 directions of body plane angular motion—pitch, roll, and yaw—representing movements in the sagittal, frontal, and transverse planes, respectively (2). Thereby, correlations between the transformed canal vestibular ocular reflex (VOR) gains with balance control weakness in, for example, the roll plane (2) could be examined. In short, the fundamental first step measuring the effect of a peripheral vestibular disease such as unilateral vestibular neuritis (UVN) on weakened superior and inferior vestibular nerve afferent gains from the lateral, anterior, and posterior canals could be quantified (2, 3). Similarly, the effect of a surgically imposed posterior canal lesion aiming to reduce intractable benign positional vertigo (4) could be followed in either canal or body planes.

One of the questions arising with vestibular UVN is whether both branches, posterior and inferior, of the vestibular nerve are equally affected by the disease. In 98 and 91%, respectively, of the 43 cases Taylor et al. (3) studied, the acute VOR gains 10 days within symptoms onset were pathological for the lateral and anterior canals (median deficit side gains were 0.4 and 0.39, respectively). In contrast, only 39% of the posterior canal deficit gains were pathological with a median gain of 0.67. Similar gain values were obtained within 5 days of acute UVN onset by Allum and Honegger (2) (mean gains 0.4, 0.44, and 0.69 for lateral, anterior, and posterior deficit side gains). There have been two explanations for this difference between posterior canal gains compared to the lateral canal gains. Firstly, it has been argued that the anatomical characteristics, a longer bony tunnel and more bony spicules make the superior vestibular nerve more susceptible to entrapment and ischemia (5). Secondly, there is evidence from slow whole body acceleration rotations that VOR gain recovery is faster in the pitch compared to the yaw (lateral canal) plane (6). In contrasting viewpoints based on vHIT tests, Büki et al. (7) argued that there was slower recovery and Lee et al. (8) faster recovery for posterior compared to lateral and anterior deficit canal gains. Thus, the question remains why the mean canal gain after onset of UVN is, on average, higher for the posterior canals.

It is well-known that commissural inhibitory projections between the vestibular nuclei act to disinhibit the contralateral vestibular nuclei activity, adding to the excitatory input from the ipsilateral side and thereby linking the bilateral vestibular nuclei together for common semicircular canal planes (9). These neural circuits are centrally adjusted to equalize gains within canal planes over time following gain reductions due to aUVN, using inputs from the cerebellum (10). A combination of this central compensation and peripheral recovery is necessary for lateral canal gains to acquire completely normal VOR gains (11). The peripheral recovery can be determined for the lateral canal using caloric tests. Thus, in cases of remaining pathological canal paresis, a range of vHIT gains are obtained in the post-acute stage depending on the amount of central compensation (12). Correspondingly, these cases with central compensation but pathological canal paresis result in normal vHIT gains which reduce the sensitivity of the vHIT for vestibular loss to 74% (12).

Unfortunately, there is, currently, no peripheral vestibular test available for the vertical semicircular canals (SSC) similar to the caloric test for the lateral canals. Such a test could determine if the combination of central compensation and peripheral recovery is different, and therefore leads to a faster recovery for the posterior canal. Thus, without a caloric test for the vertical canals, one option for an investigation of different vHIT VOR gain improvements over time for the lateral, anterior, and posterior deficit canals is to proceed under the assumption that the mix of peripheral improvement and central compensation is similar for all deficit canal gains when caused by UVN. Another option is to assume that the posterior gain is not normally affected leading to a higher gain on average.

Another way to address the question of a possible enhanced central compensation for the posterior canal is to examine the effect of a selective lesion to this canal. Such a lesion leads to permanent uncompensated VOR deficit side in the pitch direction (13) when examined at 2 months, indicating as with the lateral SSC (11) that without peripheral improvement there is a permanent deficit in posterior vHIT VOR gain. This comparison suggests that the mechanisms of central compensation might be similar for all canal planes. Another characteristic that Aw et al. (4) found in a follow-up study was that the effect of such a lesion was less severe in the pitch direction than in the roll direction and even less severe in yaw. These authors (4) attributed these effects to the geometric orientation of the SSCs in the head. That is the posterior canal is not perpendicular to the anterior canal within the labyrinth nor coplanar within the skull (14), leading to the hypothesis that vestibular nuclei neurons are probably modulated by both faciliatory and disfaciliatory activity from all canals for any head movement (15). The question arises whether this effect is due to these anatomical considerations or to the number and strengths of normal excitatory and inhibiting canal inputs contributing to pitch and roll VOR responses.

Based on the considerations above we examined changes in 3D vHIT VOR canal gains and asymmetries over initial compensation periods, which have the fastest improvement effects, in order to determine if indeed the compensation processes were different between lateral and anterior compared to posterior gains as earlier studies (2, 3) had indicated. Secondly, we examined whether these compensation processes could account for differences in roll, pitch and yaw asymmetries reported by Aw et al. (4). Our estimate of the period with fastest amount of compensation was based on our exponential modeling (12) of the improvement time course of the lateral canal gain after diagnosis of UVN. This modeling led to an estimate of 5.9 weeks for the time constant of gain improvement. That is, 63% improvement had taken place over this time period and at 3 times the time constant or 17.7 weeks 95% of the improvement. The corresponding time constant for lateral canal gain asymmetry time constant was 4.6 weeks. Thus, combining 5.9 and 4.6 into an average we decided on 5.2 weeks, after diagnosis of UVN was established, as the follow-up time. This estimate is similar to gain improvement time of 5 weeks described by Palla et al. (16). This estimate is longer than that described by Zellhuber et al. (17) for gain (1.7 weeks) but shorter than that Zellhuber et al. (17) described for symmetry improvements (7.8 weeks).

## Methods

### Subjects

The patient data used in this study was collected retrospectively at the University Hospital Basel. The study was approved by the Ethics Committee of Northwest and Central Switzerland (EKNZ), approval 2014-026, principal investigator JHJ Allum. The 20 subjects [6 females and 14 males, mean age 56.5 ± 11.0 (sd) years] with an acute unilateral peripheral vestibular deficit diagnosed as presumably due to unilateral vestibular neuritis (UVN) on the basis of a pathological lateral canal paresis to caloric testing, a pathological lateral vHIT gain on the side of canal paresis, spontaneous nystagmus beating toward the healthy ear, nausea, and the constant presence of symptoms over hours. Measurements were taken as part of the standard clinical procedure at acute onset of the UVN, on average at 4.3 ± 3.3 days, and 36.7 ± 11.8 days (5.2 or as noted below “5 weeks”) after diagnosis of UVN was established. The goal of 5.2 weeks was based on previous estimates of the time course of the lateral canal vHIT gain improvement (12). All patients were treated intravenously with methylprednisolone (125 mg Solumedrol™ per day) and then discharged 4 days after entry as an in-patient with oral medication. Written informed consent was obtained from the patients to use their data anonymously. Data from any patients with comorbid balance problems due to other causes, for example, peripheral lower leg neuropathy, was excluded from this study.

### Measurement Systems

Caloric testing: Canal paresis or unilateral weakness due to UVN was determined using a bithermal (44 and 30°C) caloric test on the same day as the 3D vHIT tests. The differences in average eye slow phase velocity (SPV) over the culmination phases of nystagmus of the 2 temperature irrigations were compared for the left and right ears. That is, if R equals the difference between the levels of SPV for the right ear irrigated with 44 vs. 30°C and L the corresponding difference for the left ear, then CP was defined as ((R-L)/(R+L))^*^100%.

To measure VOR function in response to high angular accelerations (above 2,000 deg/s^2^) a video Head Impulse Test (vHIT) system was used (ICS system from GN Otometrics, Natus Medical Inc., Taastrup, Denmark). The system was used according to the protocol described by MacDougall et al. (1) with head angular velocities reaching 100–250 deg/s by 100 ms. At least 15 head rotations with artifact-free responses in each canal plane were performed.

All vHIT tests were performed by same person (FH). During the head movements, the patient was seated with gaze fixed on a small target 3 m away. For the vertical canals, the head was first turned ~40° and then up or down head rotations were performed in the plane of the canals. Thereby, the effect of torsional eye movements on the vertical eye movement recordings was reduced (1). Sections of the data with covert saccades and artifacts were removed from the recordings prior to gain calculations by the vHIT manufacturer's software. Gains were calculated based on the quotient of the areas under the eye and head velocity impulse responses. The interval used started 100 ms prior to peak head velocity and ended when head velocity first crossed zero after this peak.

**Figure 1:**
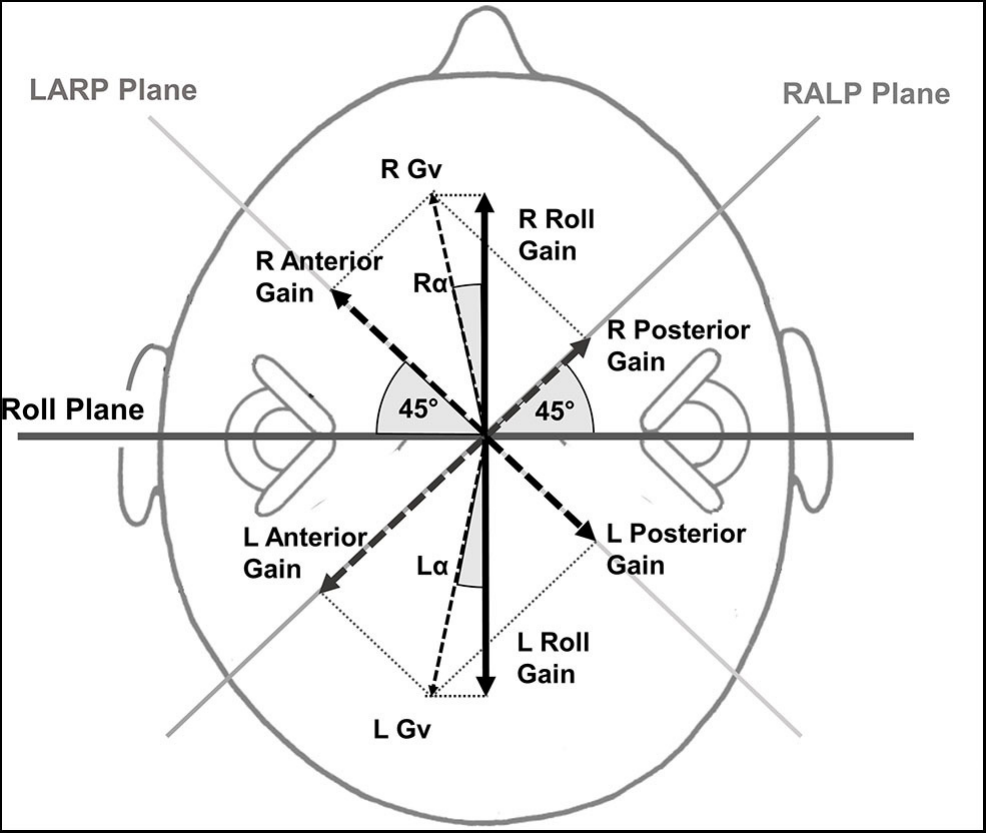
Roll gain calculation method based on the anterior and posterior vHIT canal gains. The magnitude of the anterior (*ant*) and posterior (*pos*) canal gains on one side is represented by an arrow perpendicular to the canal planes which are assumed to be at 45° to the roll plane. The corresponding vector gain Gv right (R) or left (L) in the figure has a value of $\sqrt{ant^{2}+\;pos^{2}}$. This is normalized by the value obtained if both gains were 1. That is the normalizing factor is √2 equals 1.414. The projection of the normalized Gv onto the roll axis is normalized Gv cos α where α = cos(45−tan^−1^(*ant*/*pos*)) and yields the R or L roll gain. LARP stands for left anterior, right posterior canal plane and RALP for right anterior, left posterior canal plane.

The vHIT canal gains were calculated based on head velocities imposed in the semi-circular (SCC) canal planes. Because balance control is organized along the body's roll and pitch planes (18) we converted the vertical vHIT canal gains to left and right roll, and anterior and posterior pitch gains as follows:

$$\begin{array}{l} Roll\;gain=\frac{\sqrt{ant^{2}+\;pos^{2}}}{1.414}*\cos\left(45-\tan^{-1}\left(ant/pos\right)\right) \end{array}$$

where ant is the anterior canal gain, pos is the posterior canal gain on the same side. The derivation of this equation is shown for right and left roll gain in Figure 1. The anterior pitch gain was calculated in a similar matter by combining the right and left anterior gains, likewise for the posterior pitch gain. Anterior pitch gain was always assumed to be deficit gain direction as the anterior canal gain was less, on average, than the posterior canal gain on the deficit side (see Results).

### Data Analysis

The 3 canal vHIT gains and 3 yaw, pitch and roll VOR gains were compared pairwise using a paired sample *t*-test. A Bonferroni correction was not applied to significance values quoted for the 3 comparisons in the results section because when a significance was present, its corresponding *p*-value was always <0.0001.

## Results

### Canal Gain Changes

**Figure 2:**
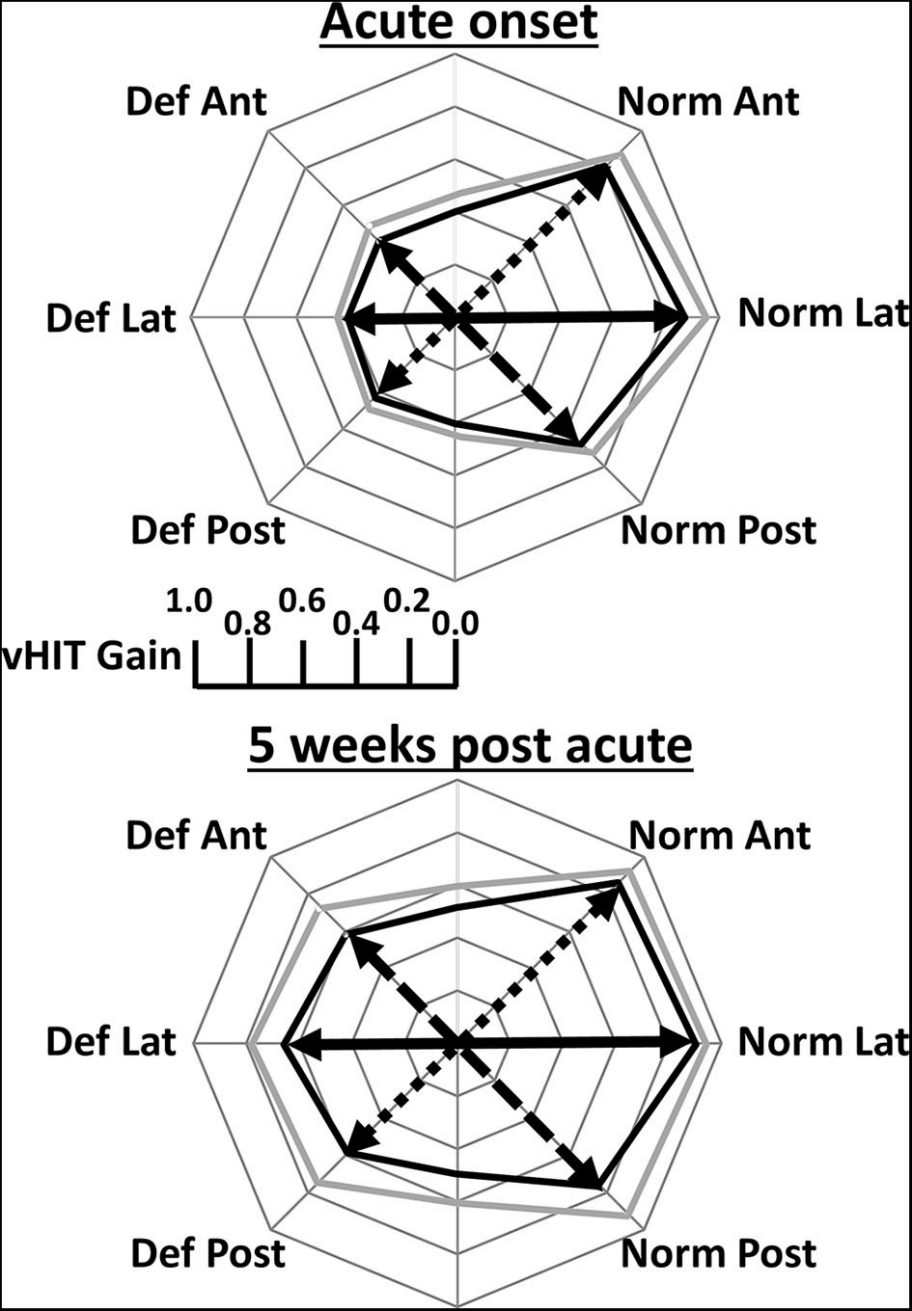
Polar plots of mean lateral (Lat), anterior (Ant), and posterior (Post) canal VOR gains obtained at acute onset of vestibular neuritis and 5 weeks later. Deficit (Def) side gains are plotted on the left and normal (Norm) side gains on the right. In contrast to Figure 1, and for ease of understanding, the gain values are represented by an arrow in the plane of the canal. The posterior Def gains are only for gains <5% lower limit of healthy controls (0.6). The mean gain values are displayed by the thick black line and the mean plus 2 standard errors of the mean by the thick gray line. Gains corresponding to the same canal plane for calculations of canal gain asymmetries are joined by a black full, dashed, and dotted line for Def Lat—Norm Lat, Def Ant—Norm Post, Def Post—Norm Ant, respectively. Exact values of the gains are provided in Table 1.

Figure 2 illustrates the deficit and normal side gain changes present after onset of aUVN and 5 weeks later in form of polar plots. Table 1 provides the corresponding numerical values of the changes. At UVN onset all lateral and 18 of 20 anterior canal deficit side gains were less than the lower limit of healthy control gains reported by Pogson et al. (19) which have been copied into Table 1. A lateral canal gain <0.79 was an inclusion requirement (see Methods) in this study. The lateral canal deficit was confirmed by the canal paresis (CP) values, all of which were higher than 64% (mean 88.6 ± 3.2% sem). A different pattern of deficit gains was obtained with deficit side posterior canal gains. Eight of 20 (40%) posterior canal deficit side gains were less than the lower healthy control limit of 0.6 at acute UVN onset (mean gain 0.43 ± 0.03), whereas the other 60% had a significantly higher mean gain of 0.75 ± 0.02 (*p* < 0.00001)—see Table 1. Of the 8 patients with posterior deficit side canal gain <0.6, 7 patients had overt catch-up saccades, 3 with covert catch-up saccades in addition. The single patient without catch-up saccades had a borderline pathological gain of 0.58. We assumed this highly significant difference for posterior canal gains less and greater than 0.6 resulted from the UVN affecting the inferior vestibular nerve function (1, 3) or not, and therefore performed subsequent analyses presented in tables 1 and 2 with posterior deficit side gains split into 2 groups based on the limit of 0.6. At onset mean lateral and anterior deficit side gains, 0.41, were approximately equal to mean gain (0.43) of those posterior canals with gains <0.6 (see Table 1). At 5 weeks deficit side gain was approximately constant (0.61) across canals and therefore showed no significant differences (Table 1). Over the 5 weeks from UVN onset the lateral, anterior, and posterior (onset gain <0.6) deficit side gains increased significantly (*p* ≤ 0.05, Table 1). The mean gain (0.75) of the posterior deficit side gains >0.6 was unchanged (Table 1).

**Table 1:** Lateral, anterior, and posterior mean canal gains ± standard error of mean for 20 patients at acute onset of unilateral vestibular neuritis (UVN) and 5 weeks later for deficit (Def) and normal side canal plane sides.

**Canal and status**	**Lateral Def (*N* = 20)**	**Anterior Def (*N* = 20)**	**Posterior Def (*N* = 8 < 0.6)**	**Posterior Def (*N* = 12 > 0.6)**	**Differences between canal gains**
Acute	0.41 ± 0.03*	0.41 ± 0.04*	0.43 ± 0.03*	0.75 ± 0.02	*different from posterior *N* = 12, *p* ≤ 0.002
Range, (number above lower HC limit)	0.2–0.76 (0)	0.12–0.81 (2)	0.23–0.51 (0)	0.63–0.84 (12)	
Differences acute and 5 weeks	↕*p* = 0.0006	↕*p* = 0.03	↕*p* = 0.05	↕*p* = ns	
At 5 weeks	0.65 ± 0.06	0.59 ± 0.07	0.58 ± 0.08	0.75 ± 0.05	No significant differences
Range, (number above lower HC limit)	0.28–1.11 (5)	0.13–1.15 (6)	0.19–0.81 (2)	0.53–0.88 (11)	
% change from acute	58.5%	37.2%	34.9%	0%	
**Canal and status**	**Lateral normal (*****N*** **=** **20)**	**Anterior normal (*****N*** **=** **20)**	**Posterior normal (*****N*** **=** **20)**	**Differences between canal gains**	
Acute	0.88 ± 0.03*	0.81 ± 0.03*	0.68 ± 0.02	*different from post *p* ≤ 0.0006	
Differences acute and 5 weeks	↕ns	↕ns	↕*p* = 0.007		
At 5 weeks	0.91 ± 0.02*	0.87 ± 0.03*	0.76 ± 0.08	*different from post *p* ≤ 0.0015	
% change from acute	4.5%	6.1%	11.8%		
Healthy control gains (*N* = 80)	0.97 ± 0.01	0.89 ± 0.01	0.78 ± 0.01		
Lower limit 95% confidence intv	0.79	0.71	0.60		

*The statistically significant t-test differences (N = 20) between different canal gains are listed in the rightmost column. The p-values are uncorrected for 3 comparisons (when Bonferroni corrected for 3 comparisons, most of the p-values are still significant). The level of significance for the difference in gain for the same canal gain at UVN onset and 5 weeks later are provided in table rows 4 and 10. The values for healthy controls (HC) are taken from Pogson et al. (19) [Gain calculation as in this report: over 60 ms prior to peak head velocity to the first head velocity zero crossing after the peak (1, 20), head velocities <200 deg/s, mean age 47 years, left and right canal gains pooled.]. The lower limits of the HC 95% confidence interval calculated from the data of Pogson et al. (19) is similar that obtained by McGarvie et al. (21). Row 3 indicates the range of gains and the number of cases with deficit side gains exceeding these limits in the acute state and row 6 provides the same information for gains at 5 weeks. As 12/20 of the posterior deficit (Def) side gains were greater than the HC limit of 0.6, deficit side (Def) canal gains are given separately for the 12 cases with posterior gains greater and for the 8 cases with posterior gains less than this HC limit. This separation was not done for the normal side posterior gains as there were no differences between mean normal side gains when the corresponding posterior deficit gains were greater than or less than 0.6*.

When the vHIT gain changes between aUVN onset and 5 weeks were examined separately for those patients with (*N* = 10) and without (*N* = 10) almost complete caloric paresis and no recovery (no recovery was defined as a CP value > 90% and remaining greater the 90% at 5 weeks), the change in deficit side lateral canal gain between UVN onset and 5 weeks was different: 0.06 for those without recovery (8 remaining at a CP of 100%), and 0.38 for those with recovery (*p* = 0.0013). Similar results have been obtained from another group of UVN patients examined at 6 weeks (11). There was a trend for deficit anterior gains to be larger for those patients with CP recovery, 0.21 increase compared with 0.08 for no CP recovery patients, but this difference was not significant. For the posterior deficit canal gain there was no observable difference in the gain changes between those with and without CP recovery.

The normal side gains also increased over 5 weeks. However, the change was only significant for the posterior canal normal side gain (*p* = 0.007). The resulting normal side canal gains at 5 weeks were, however, similar to those of healthy controls for whom gains for the posterior canal are less than those of lateral and anterior canal (see Table 1). Thus, as documented by percentage changes from onset gains listed in Table 1 and illustrated in Figure 2, the pattern of canal gains changes following onset of aUVN appears to be similar across canals.

**Figure 3:**
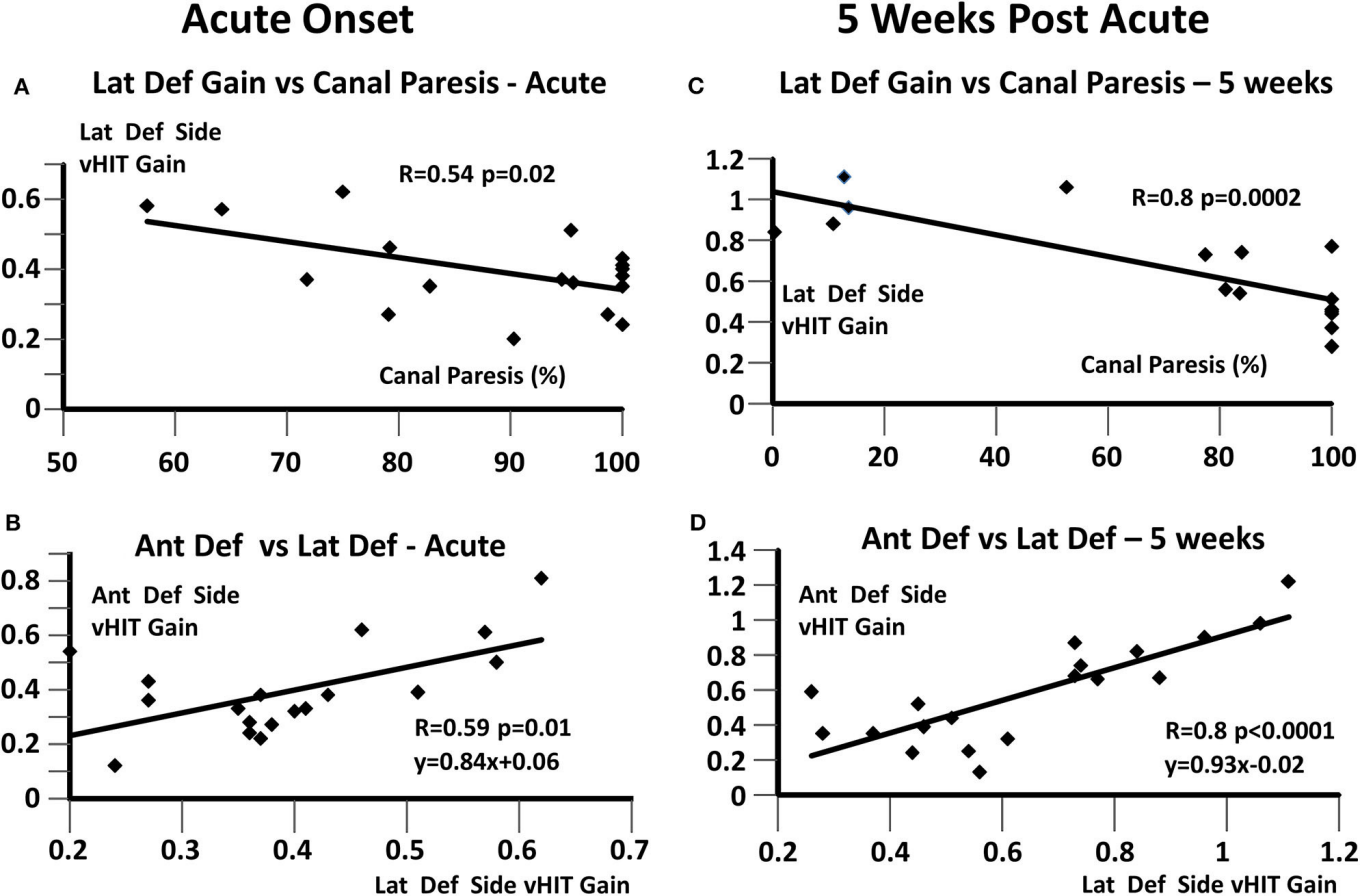
Regressions between deficit side canal vHIT gains and canal paresis, based on values at onset and 5 weeks later. **(A,C)** Lateral deficit side vHIT gain and canal paresis; **(B,D)** Anterior and lateral vHIT deficit side gains. The regression coefficient R and its significance are listed in each plot. In **(B,D)**, the linear regression equations are also provided.

The results provided in Table 1 indicate unchanged interrelationships between canal gains following UVN. We examined these relationships using linear regression techniques proceeded by an examination of the strengths of the relationship between lateral deficit side canal gain and canal paresis in the acute stage and at 5 weeks (Figures 3A,C, respectively). The relationship was stronger (*R* = 0.54 vs. 0.8) at 5 weeks due to the cases that recovered normal peripheral function. When this data was pooled, the regression indicated a significant correlation (*R* = 0.73) similar to that reported previously (*R* = 0.71) for a different group of UVN patients (12). The correlation between anterior and lateral deficit side gains also indicated a significant (*p* ≤ 0.01) almost 1:1 relationship (acute: y = 0.84x + 0.06; *R* = 0.59, 5 weeks: y = 0.93x – 0.02) between the strengths of these gains (Figures 3B,D). The correlation between posterior gains (<0.6 at onset) and lateral deficit side gains (pooled acute and 5 weeks data) was, however, weaker (*R* = 0.61), less significant (*p* = 0.03) and dominated by a constant factor (y = 0.44x + 0.30). The weaker correlation was presumably due to the smaller number of data points.

### Canal and Body Plane Asymmetries

**Figure 4:**
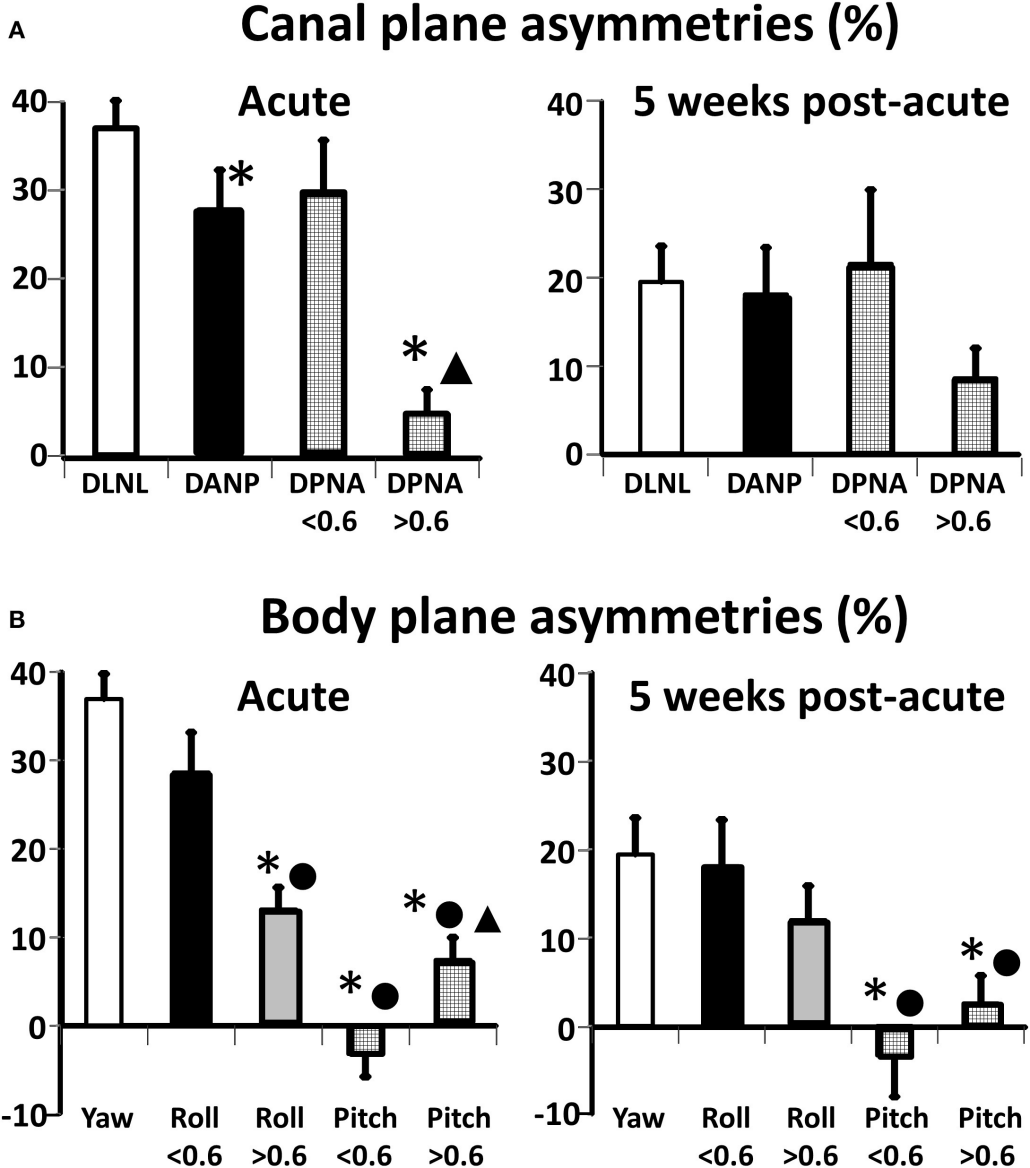
Improvements in canal **(A)** and body **(B)** plane asymmetries for acute UVN patients from acute onset (left panels) to 5 weeks later (right panels). The height of the column represents the mean value of the asymmetry and the vertical bars on the columns the standard error of the mean. The following abbreviations have been used to describe the canal planes: D, deficit; N, normal; L, lateral; A, anterior; P, posterior. For the canal plane asymmetries * indicates a significant difference with respect to DLNL asymmetry (*p* < 0.02), ▴ indicates a significant difference (*p* = 0.02) with respect to DPNA asymmetry when DP gains are <0.6. For the body plane roll and pitch asymmetries * indicate at aUVN onset a significant difference (*p* ≤ 0.0008) with respect to yaw, values of acute onset when Pos Def gain is < 0.6 between roll and pitch when Pos Def gain is >0.6 (*p* < 0.002), and between roll onset values when Pos Dif gain is >0.6 and <0.6. ▴ represents a difference between acute pitch asymmetry values when Pos Def gain was <0.6. For the 5 week values * represents pitch values less than yaw and • pitch values less than roll (*N* = 8). Note that based on the anatomical orientation DLNL, asymmetry is the same as that of yaw.

Some significant differences in canal plane asymmetries were observed with acute onset of UVN (Table 2 and Figure 4A). The asymmetry of the deficit-lateral/normal-lateral plane (DLNL) was largest and different (*p* = 0.02) from that of the deficit-anterior/normal-posterior (DANP) plane, but not that of the deficit-posterior/normal-anterior (DPNA) asymmetry when only posterior deficit gains <0.6 were considered. The DPNA asymmetry for deficit posterior gains <0.6 was significantly larger than that of the DPNA plane for deficit posterior gains >0.6 (*p* = 0.002)—see Table 2. In contrast, there was no difference in these asymmetries 5 weeks after UVN onset, when only posterior deficit gains <0.6 were considered. All asymmetries were ~20% (except the DPNA asymmetry for deficit posterior gains >0.6, 8.5%) with a gradation in the changes from the onset stage with the DLNL change being the most significant (*p* = 0.0008) and the change for DPNA (deficit posteerior gain ≤0.6) being not significant.

**Table 2:** Canal plane (upper table) and body plane (lower table) mean gain asymmetries ± standard error of mean at acute onset of unilateral vestibular neuritis (VN) and 5 weeks later.

**Canal plane and status**	**Def Lat–Normal Lat (*N* = 20)**	**Def Ant–Normal Post (*N* = 20)**	**Def Post (<0.6)–Normal Ant (*N* = 8)**	**Def Post (>0.6)–Normal Ant (*N* = 12)**	**Differences between asymmetries**	
Acute	36.9 ± 2.8%	27.7 ± 4.3%*	29.7 ± 5.6%	4.6 ± 2.5%*▴	Different from *DLNL *p* ≤ 0.02 ▴DPNA(*N* = 8) *p* = 0.002	
Differences acute and 5 weeks	↕*p* = 0.0008	↕trend	↕ns	↕ns		
At 5 weeks	19.4 ± 4.1%	18.0 ± 5.3%	21.4 ± 8.4%	8.5 ± 3.4%	No significant differences	
**Body plane and status (*****N*** **=** **20)**	**Yaw (*****N*** **=** **20)**	**Roll (*****N*** **=8) Post Def<0.6**	**Roll (*****N*** **=1 2) Post Def>0.6**	**Pitch (*****N*****=** **8) Post Def<0.6**	**Pitch (*****N*** **=** **12) Post Def>0.6**	**Differences between asymmetries**
Acute	36.9 ± 2.8%	28.6 ± 4.5%	13.1 ± 2.5%*•	−3.2 ± 3.3%*•	7.4 ± 2.7%*•▴	Different from *Yaw *p* ≤ 0.0008 •Roll *N* = 8, *p* ≤ 0.01 ▴Pitch *N* = 8, *p* = 0.03
Differences acute and 5 weeks	↕*p* = 0.0008	↕trend	↕ns	↕ns	↕ns	
At 5 weeks	19.4 ± 4.1%	18.1 ± 5.2%	12.0 ± 3.8%	−3.3 ± 5.0%*•	2.6 ± 3.1%*•	Different from *Yaw *p* ≤ 0.0002 •Roll *N* = 8, *p* ≤ 0.002

*The statistically significant paired t-test differences between different canal and body plane gain asymmetries are listed in the rightmost column. The difference in gain asymmetry for the same canal or body plane at VN onset and 5 weeks later are provided in table rows 3. As 12/20 of the posterior Def gains were greater than the HC limit of 0.6, Canal plane deficit posterior- normal anterior, roll and pitch asymmetries are provided for these 12 cases separate from the 8 cases with gains less than this HC limit. Anterior pitch gain was always assumed to be deficit gain direction even though the anterior canal gain was only marginally less than the posterior canal gain on the deficit side (see Results). Lat stands for lateral, Ant for anterior, Post for posterior, Def for deficit, DLNL for deficit lateral—normal lateral, DANP for deficit anterior—normal posterior, and ns for not significant. The p-values in the right-most column are uncorrected for 3 comparisons (when Bonferroni corrected for 3 comparisons, the p-values are still significant)*.

A similar pattern of asymmetry values was not present for the body axis asymmetries acutely (Table 2 and Figure 4B). These differences are best understood in conjunction with the polar plots of Figure 2. Pitch asymmetries were less than those of yaw (*p* ≤ 0.0008), regardless of whether the deficit posterior canal onset gain was less than or greater than 0.6. This may be understood by considering the combination of canal gain directions entering the pitch asymmetry calculation, deficit anterior and normal anterior for forward pitch, and deficit posterior and normal posterior for backwards pitch. However, at acute UVN onset the pitch asymmetry was different when asymmetries for deficit posterior gains greater and less than 0.6 were considered (Table 2 and Figure 4B). A similar effect was seen for roll (Table 2). At 5 weeks yaw (*p* = 0.0008) and roll (trend) asymmetries decreased. Pitch asymmetries at 5 weeks were still considerably less than those of yaw and roll (*p* ≤ 0.002).

## Discussion

In this study, we demonstrated that VOR gain improvements are similar across all 3 canal planes, lateral, anterior, and posterior, following onset of unilateral vestibular neuritis (UVN). Because the deficit side posterior canal nerve was less often affected by UVN than the deficit side lateral and anterior canal nerves it was necessary, for the purposes of deficit gain calculations, to separate the deficit posterior canal gains into those above and below a lower normal limit of 0.6 (19). This resulted in 40% of the cases being classified as having a pathological posterior canal gain in addition to the pathological lateral canal gain, exactly equal to the percentage noted by Taylor et al. (3). When considered under this posterior canal gain criterion of 0.6, there were no statistically significant differences between the onset deficit side gain values across canals and no statistically significant differences between the 5 week deficit side gain values across canals. However, the deficit side gain change between onset and 5 weeks was greater and therefore more significant for the lateral canal and least significant for the posterior canal. Conversely, the normal side gain changes were most significant for the posterior canal and least significant for the lateral canal. This pattern of deficit and normal side gain changes led to similar canal plane asymmetries across canal planes being on average 31.4% at aUVN onset and 19.6% 5 weeks later. These similarities across canals suggest common gain improvement processes across canals as we discuss in detail below. Both peripheral improvement (if some recovery is present) and central compensation can contribute to the gain improvements.

The similarities in canal plane gain asymmetries does not imply that these similarities transfer to body plane axes along which balance corrections are programmed by the CNS as demonstrated by Grüneberg et al. (18). The combination of 2 deficit vertical canal measures for one direction of roll and 2 normal vertical canal measures for the other direction of roll compared to a combination of 1 deficit side and 1 normal side measures for each direction of pitch leads to lower pitch than roll asymmetries regardless of whether only pathological posterior deficit canal gains (<0.6) are considered. If balance corrections are modulated by canal inputs, our results imply that balance instability during stance and gait tasks should be in the order of least for pitch, greater for roll, and greatest for yaw. Further that body sway amplitudes during balance tasks will be less or more correlated, respectively, with the corresponding VOR gain asymmetry. For quiet stance and gait tasks pitch instability is greater than roll but not correlated with VOR pitch asymmetries (2). In contrast, roll plane balance instability during gait trials is highly correlated with VOR roll plane asymmetry (2). The current results suggest a similar correlation as that of roll should be present for yaw.

Similar gain changes for anterior and posterior deficit side gains compared to those of the lateral canal were observed in the UVN patients. Does this imply that the mix of peripheral recovery and central compensation was common across deficit canals? For the lateral canals, this mix varies, leading to a lower average gain change of 0.24 across all cases, to a greater average gain change of 0.38, for cases for which some peripheral recovery was present. Similar results were observed by Allum et al. (11). From these results, one can estimate the contribution of peripheral recovery to the improvement in VOR gain as ~60% (11). Given the size of this contribution, it would appear important to develop caloric tests of peripheral vertical canal function (22, 23) in order to quantify the amount of peripheral improvement vs. central compensation. Alternatively, electrophysiological techniques measuring afferent nerve responses could be developed to determine the amplitude of vestibular nerve action potentials observed when canal nerves are selectively activated (24).

A number of authors have observed, as we did, a slight increase in gain on the normal side during the post-acute compensation process (7, 16, 25). The studies of Palla and Straumann (16) and those of Fu et al. (25) were concerned with the lateral canal, but Büki et al. (7) observed this effect for all 3 canals on the contra-deficit (normal) side. Palla and Straumann (16) argued that the normal side was rapidly compensated as otherwise the lack of disinhibitory input via commissural pathways would cause a greater gain reduction. The alternative hypothesis fitting the current results is that the disinhibition is in any case limited by the high head acceleration of the vHIT (26) and the effect only becomes apparent when the vestibular nerve is cut (27) or sufficient large numbers (~70) of UVN patients are examined (Cleworth, Allum, and Honegger, unpublished observations 2020). In the current study, the number of patients was not sufficient to obtain a significant result except for the posterior normal side canal gain.

We used an exponential model (12) to estimate the change in lateral canal deficit side vHIT VOR gain over time:

(1) $$\begin{array}{r} Deficit\;side\;gain=0.82-0.29\exp\left(-t/5.88\right) \end{array}$$

where t is the time post aUVN onset in weeks. Our prior estimate for the lateral canal gain at 5.2 weeks, the average time of this study's follow-up examination, was 0.64 compared to the current result obtained of 0.65. Similar values were obtained for the vertical canal gains, 0.59 and 0.58. An identical procedure could be used to estimate canal plane asymmetry at 5.2 weeks. The estimate was 19.5% compared to the average value across canals of 19.6%. That a single model could be used to describe the findings across all 3 canal planes reinforces the viewpoint that peripheral improvement and central compensatory mechanisms are similar across these planes. We have not been able to locate similar studies examining early gain changes following onset of VN in all 3 canal planes. Most studies have been conceived with the aim of determining recovery rates. For example, Büki et al. (7) recorded the number of patients who recovered at 10 weeks post UVN onset when gains according to equation 1 above would have been within 14% of steady-state. Taylor et al. (3) traced the pattern of pathology across all canals and otoliths at UVN onset and up to 12 months later when no further increase in gain could be expected. Fu et al. (25) traced lateral canal gains at 10 days after aUVN onset and 6 months later. Their values of 0.47 at onset and 0.69 at 6 months are similar to ours of 0.41 and 0.65 given the differences in measurement times and emphasizes the advantages of early re-testing at 5–6 weeks, namely that the patient can be informed whether their improvement is following the expected time course and, if not, rehabilitatory physiotherapy could be intensified.

Although we have argued that central compensation processes underlying gain improvements following aUVN are common across canal planes, there are at least 3 limitations to be taken into in the current study. Firstly, although we obtained similar onset deficit gains for the lateral and anterior canals (0.4 and 0.44) as in our previous study with 33 subjects it is possible that using more than 20 subjects used in the current study would have brought greater significance to our results. In the previous study (2) we did not separate patients on the basis of posterior canal gains above and below the normal limit of 0.6. With this separation, the number of subject was further reduced. Also, it should be borne in mind that for the vertical canal recording the gaze was maintained eccentric at 40° in order to minimize torsional and maximize vertical eye movements (21). Consistent variations in the gaze angle could lead to gain variations. However, we recorded very similar deficit side VN onset gains for the 3 canal axes (0.41, 0.41, and 0.43). Therefore, it appears there were not any systematic differences between canal gains to indicate a too low eccentric gaze angle. Thirdly, it would have been interesting to note if cervical vestibular evoked myogenic potentials (c-Vemps) were pathological for those cases with posterior canal gains less than 0.6, as was found for 50% of these patients by Taylor et al. (3). Pathological cVemps would have confirmed the inferior vestibular nerve involvement. Thus it is a possible limitation of the current study that we did not record cVemps in these cases.

It was at first sight surprising that pitch plane asymmetries calculated from the canal gains even at 5 weeks post UVD onset were considerably less than those of roll. When, however, the orientation and number of contra-deficit vertical canal gains acting in the pitch and roll directions are considered, this result is not surprising. Examining the polar plots of Figure 2, it is very apparent that asymmetries would be greater in the roll direction.

When normal and bilateral vestibular loss patients receive perturbations to stance in the roll and pitch directions, we have argued that vestibular and proprioceptive contributions to roll and pitch balance corrections need to be processed differently due to the timing of contributing sensory signals (28, 29). We can now add another reason for this different processing—pitch-oriented perturbations are likely to be processed more accurately in the event of vestibular loss.

The other notable aspect of the current investigation is that yaw asymmetries were equally affected as those of roll. Based on this result perhaps posturographic examinations should change their focus on balance deficits in the pitch and roll planes to yaw and roll planes, for example by investigating the balance instability in the roll and yaw planes when a person circumvents another person in a crowd (30).

## Data Availability Statement

The raw data supporting the conclusions of this article will be made available by the authors, without undue reservation.

## Ethics Statement

The studies involving human participants were reviewed and approved by Ethical committee of North-West Switzerland. The patients/participants provided their written informed consent to participate in this study.

## Author Contributions

JA conceived of the experiments, carried out the data analysis, and wrote the first draft of the manuscript. FH recorded almost all of the patient data, checked the data analysis, and corrected the manuscript. All authors contributed to the article and approved the submitted version.

## Conflict of Interest

The authors declare that the research was conducted in the absence of any commercial or financial relationships that could be construed as a potential conflict of interest.
